# Retraction: Serum Levels of 25-Hydroxyvitamin D and Functional Outcome among Postmenopausal Women with Hip Fracture

**DOI:** 10.1371/journal.pone.0219278

**Published:** 2019-07-01

**Authors:** The PLOS ONE Editors

After publication it was noted that this article [1] addresses the same research question and reports highly similar results and conclusions as a publication in the *Journal of Arthroplasty* [2]. There is substantial text overlap between the publications. The two articles were authored by different research groups and were under consideration during overlapping periods per the published article information. When asked about the similarities, the corresponding author for the *PLOS ONE* article commented that the work had been conducted in collaboration between three hospitals including the institution listed in [2], and that the authors of the two articles diverged in their work prior to publication. The *PLOS ONE* article [1] specifies that it reports a single-department study, and no collaboration or related work was declared for the *PLOS ONE* submission.

In reviewing this matter, we identified additional concerns for the *PLOS ONE* article [1]:

- The author list was changed in its entirety between submission and publication. The corresponding author has affirmed that the published list of authors is correct.
- Between submission and publication, the Methods and Ethics Statement were revised to indicate that the work had been conducted at a different hospital, yet the recruitment dates, patient numbers, and other data were not changed.
- There is substantial text overlap between [1] and other published works, including [3–7]. With the exception of [5], these articles were not cited.

In light of these issues, which include concerns about redundant publication, text overlap, and the integrity of both the study and author contributions to the work, the *PLOS ONE* Editors retract this article.

The authors did not comment on the retraction decision.

Due to similarities with previously published content [2] that is not licensed for reproduction and distribution under the terms of the Creative Commons Attribution License, Figs 1 and 2 have been removed from the retracted article.

## References

[1] Liu L-M, Wang S-H, Fu C-S, Han X-Z, Wei B-F (2015) Serum Levels of 25-Hydroxyvitamin D and Functional Outcome among Postmenopausal Women with Hip Fracture. PLoS ONE 10(1): e0116375 10.1371/journal.pone.0116375 25635882 PMC4312033

[2] Wang X-G, Yang B, Wang Y-H, Cui L-Y, Luo J-P. (2015) Serum Levels of 25-hydroxyvitamin D and Functional Outcome in Older Patients with Hip Fracture. J Arthroplasty 30(5): 891–894. (published online 12/18/2014) https://www.arthroplastyjournal.org/article/S0883-5403(14)00949-8/fulltext 10.1016/j.arth.2014.12.018 25603761

[3] Zhang W and Zhang X-A. (2014) Prognostic value of serum lipoprotein(a) levels in patients with acute ischemic stroke. NeuroReport 25(4): 262–266. https://insights.ovid.com/crossref?an=00001756-201403050-00010 10.1097/WNR.0000000000000094 24356104

[4] Fu C-W, Liu J-T, Tu W-J, Yang J-Q, Cao Y. (2014) Association between serum 25‐hydroxyvitamin D levels measured 24 hours after delivery and postpartum depression. BJOG: An International Journal of Obstetrics & Gynaecology. 122(12): 1688–1694. https://obgyn.onlinelibrary.wiley.com/doi/full/10.1111/1471-0528.13111 10.1111/1471-0528.13111 25233808

[5] Di Monaco M, Vallero F, Castiglioni C, Di Monaco R, Tappero R. (2011) Low levels of 25-hydroxyvitamin D are associated with the occurrence of concomitant upper limb fractures in older women who sustain a fall-related fracture of the hip. Maturitas 68(1): 79–82.http://www.maturitas.org/article/S0378-5122%2810%2900345-2/abstract 10.1016/j.maturitas.2010.09.001 20888157

[6] Li Y-T, Zhao Y, Zhang H-J, Zhao W-L. (2014) The Association between Serum Leptin and Post Stroke Depression: Results from a Cohort Study. PLoS ONE 9(7): e103137 10.1371/journal.pone.0103137 25061971 PMC4111552

[7] Sulter G, Steen C, De Keyser J. (1999) Use of the Barthel Index and Modified Rankin Scale in Acute Stroke Trials. Stroke 30(8): 1538–1541.http://stroke.ahajournals.org/content/30/8/1538.full 10436097 10.1161/01.str.30.8.1538

